# Choosing emergency medicine: Influences on medical students’ choice of emergency medicine

**DOI:** 10.1371/journal.pone.0196639

**Published:** 2018-05-09

**Authors:** John C. Ray, Laura R. Hopson, William Peterson, Sally A. Santen, Sorabh Khandelwal, Fiona E. Gallahue, Melissa White, John C. Burkhardt

**Affiliations:** 1 Department of Emergency Medicine, Medical College of Wisconsin, Milwaukee, Wisconsin, United States of America; 2 Department of Emergency Medicine, University of Michigan, Ann Arbor, Michigan, United States of America; 3 Department of Emergency Medicine, Virginia Commonwealth University, Richmond, Virginia, United States of America; 4 Department of Emergency Medicine, Ohio State University, Columbus, Ohio, United States of America; 5 Division of Emergency Medicine, University of Washington, Seattle, Washington, United States of America; 6 Department of Emergency Medicine, Emory University, Atlanta, Georgia, United States of America; Rice University, UNITED STATES

## Abstract

**Background:**

Relatively little is understood about which factors influence students’ choice of specialty and when learners ultimately make this decision.

**Objective:**

The objective is to understand how experiences of medical students relate to the timing of selection of Emergency Medicine (EM) as a specialty. Of specific interest were factors such as how earlier and more positive specialty exposure may impact the decision-making process of medical students.

**Methods:**

A cross-sectional survey study of EM bound 4th year US medical students (MD and DO) was performed exploring when and why students choose EM as their specialty. An electronic survey was distributed in March 2015 to all medical students who applied to an EM residency at 4 programs representing different geographical regions. Descriptive analyses and multinomial logistic regressions were performed.

**Results:**

793/1372 (58%) responded. Over half had EM experience prior to medical school. When students selected EM varied: 13.9% prior to, 50.4% during, and 35.7% after their M3 year. Early exposure, presence of an EM residency program, previous employment in the ED, experience as a pre-hospital provider, and completion of an M3 EM clerkship were associated with earlier selection. Delayed exposure to EM was associated with later selection of EM.

**Conclusions:**

Early exposure and prior life experiences were associated with choosing EM earlier in medical school. The third year was identified as the most common time for definitively choosing the specialty.

## Introduction

Every medical specialty seeks the best and the brightest students. How, when and why students chose a specialty provides helpful guidance on how best to achieve these goals.[1–3] The Emergency Medicine specialty provides a good example of the interplay between competitiveness and fit, as it seeks to offer desirable, lifelong careers to the top medical students. In 2015 over 1800 medical students entered into residency in Emergency Medicine (EM). This number has increased by over 6% annually.[4] Recent literature regarding the EM career selection process has focused on differences in future income and lifestyle preferences,[5–7] but there is little information regarding how students’ past experiences and exposure to EM in medical school influence specialty selection. Given that EM rotations generally occur later in a medical student’s career than many other specialties (internal medicine, general surgery) consideration of a career in the specialty may not occur to some students until after their options have become more limited. Therefore, improved understanding of the decision process of specialties with more limited early clinical exposure, such as EM, would be of practical importance to program directors, clerkship directors, and other policy makers for refining student recruitment approaches to the specialty.

When medical students make their specialty choices, their decisions are often based on limited factors, such as clinical exposure, time spent in the desired field, and perceived likelihood of success.[8] Medical student decision-making in this setting fits a “bounded rationality” model, which acknowledges incomplete search behavior, while still viewing the decision-making as rational and systemic.[9] Bounded rationality theory also explicitly posits that individuals have variability in what they value most, both consciously and unconsciously, and thus, examination of the “rationality” of others’ decisions must take this idea into consideration. As a result of this theoretical approach, we believe medical students each attempt to maximize the things most important to them (though as external reviewers we can only infer what these values are and they may be different than our own), however, their selection approach may be further limited (or flawed) by inadequate information or inaccurate assumptions on the part of the student. Similar approaches have previously been used in the evaluation of academic medical career selection. [10] We are the first to apply this approach to medical specialty choice but it has been extensively used in other models of student decision making in higher education. Building on work from this related field, we examined pre-medical school exposure to EM, participation in EM clerkships, EM-specific mentorship, and the timing of medical school exposure to the EM field as influences on career selection. This study hypothesizes that earlier exposure and more positive experiences for medical students who ultimately chose EM have meaningful impacts on the decision-making process.

## Methods

### Study design/Setting

We performed a cross-sectional, web-based survey of US medical students (M.D. and D.O.) graduating between Dec 2014 and June 2015 who applied in EM to identify influences on career selection. For content validity, the survey was designed after a literature review, expert opinion input (e.g. medical school administrators, residency and clerkship directors, and faculty) and input from fourth year students and residents. The items were not cross validated as we were unable to identify any appropriate validated tool available for our use. To optimize content, response process, and internal structure evidence, we created our survey instrument using the Delphi method. This included testing among the authors for item generation, survey functionality, matching of item content to the construct, optimal item phrasing, and overall quality control. For response process validity, the survey was piloted and revised. A copy of the survey is available in S1 File.

### Selection of participants

The survey was sent by E-mail using Qualtrics^™^ in the first week of March 2015 to 1372 students who applied to at least one of four different EM residency programs (approximately 64% of US medical school graduates applying to EM). As geography often influences residency choice, participating institutions were chosen from the Midwest (2 programs), South, and West.[11]. Survey participants remained anonymous. The applicants’ email addresses were obtained through the Electronic Residency Application Service (ERAS) and use of applicant email addresses for research purposes was reviewed and approved by its parent organization, the AAMC. Email addresses were available in the raw output provided by Qualtrics but were not included in any analytical process. Residency selection decision-makers were not provided identifiable information. The survey was performed intentionally after the Match list submission date but prior to Match Day in order to minimize any risk associated with identified responses. Analysis was performed by a separate Emergency Medicine faculty who was not involved in residency selection operations (J. Burkhardt). The data set was de-identified when data analysis was performed. No individual specific information was provided to anyone outside of the primary data analyst. Student responses were confidential. Reminders were sent weekly to non-responders for three consecutive weeks.

### Outcomes

We created multiple binary logistic regression models to examine different aspects of the student specialty selection process. The outcome variable for all models was a categorical variable on when students decided on EM (Before M3, M3, M4 or after). Individual independent factors included timing of first exposure to EM, nature of first meaningful exposure, consideration of alternative medical specialties and self-reported factors related to why the student chose EM. The preferred models were based on our theoretical framework and model fit specifications.

### Analysis

Descriptive analyses and multinomial logistic regressions were performed on the obtained responses using STATA 12.^™^ All survey responses that included values for all model factors were used in the analysis. No data imputation was used for incomplete survey responses.

The study was determined to be exempt under exemption 2 by the IRB review committee noting that the study provided no more than minimal risk to the participants.

## Results

The survey was emailed to 1372 US senior applicant students to EM in 2015.[4] Demographic responses are summarized in Table 1 and compared with AAMC data from the 2014–15 application season.[12] Survey responses for questions 1–8 are available in S2 File. Over half of students (58%) had EM experience prior to medical school, yet few had decided on EM when starting medical school (8%). The three most important ranked reasons for choosing EM were variety in clinical encounters, work/life balance, and perceived job satisfaction, respectively.

**Table 1:** Survey demographics as compared to US medical school graduates who applied to EM.

Survey Responses	AAMC EM application data+
**Gender**	**Gender**
63% Male (485)	67% Male (1417) *
37% Female (288)	33% Female (699)
**Racial Background**	**Racial Background**
67% White (565)	60% White (1354) **
10% Asian /Pacific Islander (83)	20% Asian / Pacific Islander (441)
4% Hispanic (35)4% Black/AA (32)	9% Hispanic (213)
15% Other or no response (125)	8% Black/ AA (182)
	3% Other or no response (59)
**Med School Location**	
29% South (225)	
25% Northeast (196)	
18% West (140)	
27% Midwest (211)	

Chi Squared Goodness of Fit Test *P Value <0.05; ** P Value <0.0001 +AAMC Data used for general comparison of population demographics and includes both US seniors and some other applicants. Not meant to exactly match survey applicant characteristics but to allow for consideration of demographic sample bias. Survey Respondents (n = 793); AAMC applicants (n = 2116).

The results from the binomial regression analyses including (i) alternative specialty consideration and (ii) prior experiences influencing specialty decision, are depicted in Tables 2 and 3 respectively. Of the students who decided on EM, 110 (13.9%) decided prior to Year 3, 399 (50.4%) decided during Year 3, and 282 (35.7%) decided in Year 4 or later. The top 5 most common specialties considered as either a student’s first or second alternative were Internal Medicine, General Surgery, Anesthesiology, Family Medicine, and Pediatrics. An interest in an alternative specialty significantly (p<0.05) decreased the likelihood of earlier selection of EM (Table 3). Early exposure, presence of an EM residency program, prior employment in the ED, previous experience as a pre-hospital provider, and completion of a Year 3 clerkship were associated with earlier selection of EM. Delayed exposure to EM until year three was associated with later selection of EM (Table 2).

**Table 2:** Does consideration of other medical specialties affect decision timing on EM?

	Decided on EM during_M3		Decided on EM during M4 or still deciding	
VARIABLES (1 = high interest)	Relative Risk Ratio	95% Confidence Interval	Relative Risk Ratio	95% Confidence Interval
Interest in Anesthesia Rank	0.98 (0.01)	0.95–1.01	0.98 (0.01)	0.96–1.01
Interest in Family Medicine Rank	0.96* (0.02)	0.93–0.99	0.95** (0.02)	0.92–0.98
Interest in Internal Medicine Rank	0.96*** (0.01)	0.93–0.98	0.94*** (0.01)	0.92–0.97
Interest in Orthopedic Surgery Rank	0.98 (0.02)	0.95–1.02	0.99 (0.02)	0.96–1.03
Interest in Pediatrics Rank	0.97* (0.02)	0.93–0.99	0.97 (0.02)	0.94–1.00
Interest in General Surgery Rank	0.96** (0.01)	0.93–0.99	0.96*** (0.01)	0.93–0.98
Interest in Radiology Rank	0.98 (0.03)	0.92–1.05	0.95 (0.03)	0.89–1.01

Outcome Comparison is Deciding before M3; SE in parentheses;

*** p<0.001,

** p<0.01,

*p<0.05; Area under ROC .71

**Table 3:** Do medical students choose EM sooner based on previous experiences?

	Decided on EM during M3		Decided on EM during M4 or still deciding	
VARIABLES	Relative Risk Ratio	95% Confidence Interval	Relative Risk Ratio	95% Confidence Interval
No affiliated EM residency program at medical school	0.63 (0.16)	0.39–1.02	0.56* (0.15)	0.33–0.95
When was your first exposure to EM				
Year 1	0.78 (0.28)	0.39–1.57	0.71 (0.28)	0.33–1.53
Year 2	1.16 (0.62)	0.4–3.30	1.63 (0.91)	0.54–4.88
Year 3	11.69* (12.43)	1.46–93.91	16.57** (17.74)	2.03–135.20
Year 4	0.89 (0.80)	0.15–5.16	5.41* (4.58)	1.03–28.41
What was your first meaningful EM exposure				
Research	1.25 (0.84)	0.33–4.69	0.98 (0.72)	0.24–4.09
Employment in ED	0.70 (0.24)	0.36–1.36	0.44* (0.18)	0.20–0.97
Required Clerkship in EM	2.01 (1.29)	0.58–7.04	2.74 (1.77)	0.77–9.72
Pre-hospital Provider	0.45* (0.15)	0.24–0.85	0.63 (0.22)	0.31–1.25
Personal/Family cared for in ED	1.60 (0.94)	0.50–5.06	1.68 (1.04)	0.50–5.67
Other	0.95 (0.43)	0.40–2.30	0.79 (0.39)	0.30–2.07
Did have a Year 3 Rotation	1.42 (0.33)	0.90–2.23	0.45** (0.12)	0.27–0.75

Outcome Comparison is Deciding before M3; Independent Variable Comparison Group is applicant at school with EM program, first experience prior to medical school, first meaningful exposure was clinical shadowing; SE in parentheses;

*** p<0.001,

** p<0.01,

* p<0.05; Hosmer-Lemeshow Not significant using deciles, Area under ROC .71

## Discussion

Medical specialty choice involves a complex decision-making process[13,14] containing many influencing factors for medical students. We approached the question of EM specialty selection using a model grounded in theory to inform the factors we considered and we believe a similar approach would also be possible across other medical specialties. Our study illustrates that there are several key factors in the pre-medical and medical school experiences in motivating medical students’ choice of a career in EM. For example, nearly 75% of EM bound students come from medical schools with an affiliated EM residency program. Similarly, early EM exposure was common (50% of the respondents were exposed to EM before they enrolled in medical school). This is in keeping with other specialties, such as Orthopedics[3], Family Medicine[2], and Psychiatry[1] have reported significant correlation between specialty selection and pre-clinical exposure to the field.

Most of our respondents decided on EM by the end of M3 (64%). The timing of this decision is important to note, as EM and many other medical specialties have their rotation located in the fourth year of medical school and thus many students may not have significant specialty contact until after their decision has been made.[15] These findings speak to the potential benefit of providing early clinical exposures in the first two years to specialties with later and/or elective clinical rotations as well as the development of core clerkship during the third year. For example, many surgical sub-specialties such as Orthopedics and Urology may not have required clinical rotations and therefore must be more pro-active in increasing student-faculty interaction.

Advocacy for increasing early exposure to fields like EM is not about increasing application numbers; many fields with late rotations have more than sufficient applicants to fully match and be competitive.[16] By advocating for earlier, meaningful exposures to specialties that have later rotations, students are armed with critical information about the career path that might make them happiest. Faculty in Emergency Medicine, as well as other specialties, have all mentored students that wanted to change their specialty focus but arrived at the decision too late to pursue it. In the end, medical educators want all students to be fulfilled, interested, engaged and successful in their careers. Utilizing the evidence on career selection can help them do that.

When asked which other specialties EM-bound students considered, Internal Medicine, General Surgery, and Anesthesia were most common. Accordingly, an ED relationship with such other desirable specialties is a potentially powerful recruitment tool. Cooperating in this way can be mutually beneficial in combating negative stereotypes that students might have about certain specialties.[17] Negative stereotypes have been shown to decrease applications to certain specialties in the resident match.[2,18,19] In the specific case of EM, actively demonstrating to students the vital role that EM plays as an essential partner with to Internal Medicine, Surgery, and Pediatrics is a key consideration in EM recruitment.

To date no study has specifically approached the question of EM specialty selection using this type of theoretically derived model, and we believe our model to be generally applicable to similarly represented specialties in academic medicine. This study illustrates the individual impact that pre-medical and medical school experiences have on when students choose a medical field, as well as, moving the underlying theoretical construction of specialty selection forward. This type of inquiry provides essential information, as many medical schools have begun to re-evaluate and restructure their current medical school curricula.

Several limitations of the study were identified. First, the survey was distributed to fourth year medical students who had already applied to an EM residency, thus our cohort only represents students who selected EM, and does not represent students considering EM who ultimately chose not to apply. Thus our study does not explicitly test the correlation between early EM exposure and choosing another specialty, and as it is limited to EM applicants it does not test if applicants to other specialties behave differently. The choice of this sub-population, while limiting what can be said about the general population, was intentional. Our research question was focused on the factors that impact the timing on a decision to enter EM, not what factors are important in choosing EM. Both are important questions, but in focusing on the former we were able to ask more specific questions in our survey. Additionally the sample population was drawn from applicants who applied to University of Michigan, University of Washington, Emory University, and Ohio State University. This does not represent all geographical regions namely the Northeast and Southwest. The absence of these areas could introduce bias to the sample populations as the values of the applicants from different regional locals certainly could be different to affect their decisions and timing of decisions. Second, the survey was distributed after the students had submitted their rank lists to minimize potential response bias for fear of negative repercussions, but this may still exist. Finally, a statistically significant difference exists in the demographic makeup of our sample cohort compared with the AAMC reported data for EM matriculating US medical school graduates for both gender and racial background demographics.

As a next step, we would suggest examining if the timing of specialty selection is dissimilar across fields and how interest in other specialties impact the time to decision in fields other than EM. The authors did initially attempt to fit a larger model including more specialty choices but refined the model based on several considerations. First, limiting the variables improved model fit. Second, many of the variables had too few responses in some of the outcome categories that assumptions of logistic regression were not met. As a result of this, concavity of the model could not be achieved for the desired base outcome (deciding before M3) with this many factors. Finally, the refined model included the most salient specialties given our conceptual framework and the raw survey responses

## Conclusion

Early specialty exposure was shown to influence specialty selection, and along with specialty-specific mentorship, can play a significant role in attracting medical students. Our findings are consistent with a student decision-making process that is affected by the availability of information and the experience of applicants who are making their decisions earlier in their medical career than most EM clerkships.

## Supporting information

S1 File Distributed survey. (DOCX)

S2 File Breakdown of responses to survey questions. (DOCX)
