# Supplementary material for: Choosing emergency medicine: Influences on medical students’ choice of emergency medicine
Source: PLoS One. 2018 May 9;13(5):e0196639. doi: 10.1371/journal.pone.0196639 (PMC5942813; doi:10.1371/journal.pone.0196639)
Supplement: S1 File — (DOCX) [file pone.0196639.s001.docx]

Appendix 1: Distributed Survey

| 1. Does your Medical School have an affiliated Emergency Medicine Residency? |
| --- |
| ☐ Yes |
| ☐ No |
| ☐ Unsure |
| 1. When were you first exposed to Emergency Medicine? |
| ☐ Prior to start of Medical School |
| ☐ M1 |
| ☐ M2 |
| ☐ M3 |
| ☐ M4 |
| 1. What was the nature of your first meaningful exposure to Emergency Medicine? |
| ☐ Clinical Shadowing |
| ☐ Research |
| ☐ Employment in the ED (e.g. Nurse, Tech, Clerk, Scribe etc.) |
| ☐ Required Clerkship or Elective in EM |
| ☐ Pre-Hospital (First Responder, EMT, Paramedic, Firefighter etc.) |
| ☐ Personal or family medical encounter |
| ☐ Other |
| 1. Did you take an Emergency Medicine Clerkship? |
| ☐ Elective M3 |
| ☐ Required M3 |
| ☐ Elective M4 |
| ☐ Required M 4 |
| ☐ I did not participate in an Emergency Medicine Clerkship |
| 1. Pick and rank all other residency choices that you considered prior to deciding on your final specialty. (If you considered a different residency program, drag that program into the box in the order of importance) |
| ☐ Anesthesia |
| ☐ Dermatology |
| ☐ Family Medicine |
| ☐ Internal Medicine |
| ☐ Neurology |
| ☐ OB/GYN |
| ☐ Ophthalmology |
| ☐ Orthopedic Surgery |
| ☐ Otolaryngology |
| ☐ Pathology |
| ☐ Pediatrics |
| ☐ PM&R |
| ☐ Psychiatry |
| ☐ Radiology |
| ☐ Radiation Oncology |
| ☐ General Surgery |
| ☐ Urology |
| ☐ Other |
| ☐ Cardiovascular Surgery |
| ☐ Vascular Surgery |
| ☐ Plastic Surgery |
| ☐ Neurosurgery |
| ☐ I did not consider other fields |
| 1. When did you definitively decide on Emergency Medicine as a specialty? |
| ☐ Prior to start |
| ☐ M1 |
| ☐ M2 |
| ☐ M3 |
| ☐ M4 |
| ☐ I am still deciding |
| 1. Please check all previous experiences you had prior to final specialty selection. |
| ☐ Shadowing opportunity |
| ☐ Required clerkship |
| ☐ Elective rotation |
| ☐ Worked on original research in field |
| ☐ Published research in field |
| ☐ Family member in field |
| ☐ Mentor in field |
| 1. Please rank the following in order of importance in choosing your specialty. Only include the items that you consider important. |
| ☐ Perceived job satisfaction |
| ☐ Financial compensation |
| ☐ Intellectual compensation |
| ☐ Demands on family |
| ☐ Opportunity to teach |
| ☐ Work life balance |
| ☐ Variety in clinical encounters |
| ☐ Professional prestige |
| ☐ Opportunity to do research |
| ☐ Patient population served |
