# Supplementary material for: Choosing emergency medicine: Influences on medical students’ choice of emergency medicine
Source: PLoS One. 2018 May 9;13(5):e0196639. doi: 10.1371/journal.pone.0196639 (PMC5942813; doi:10.1371/journal.pone.0196639)
Supplement: S2 File — (DOCX) [file pone.0196639.s002.docx]

**Appendix A: Breakdown of responses to survey questions**

| **Q1. Does your Medical School have an affiliated Emergency Medicine Residency?** | | |
| --- | --- | --- |
| Yes | | 75% |
| No | | 25% |
| **Q2. When were you first exposed to Emergency Medicine?** | | |
| Prior to the start of medical school | 58 % | |
| M1 | 12 % | |
| M2 | 6 % | |
| M3 | 16 % | |
| M4 | 8 % | |
| **Q3. What was the nature of your first meaningful exposure to Emergency Medicine?** | | |
| Clinical Shadowing | 30% | |
| Employment in the ED | 14% | |
| Pre-Hospital Employment | 18% | |
| Research | 3% | |
| Required Clerkship or Elective in EM | 22% | |
| Personal or family medical encounter | 6% | |
| Other | 7% | |
| **Q4. Did you take an Emergency Medicine Clerkship?** | | |
| Elective M3 | | 19% |
| Required M3 | | 8% |
| Elective M4 | | 46% |
| Required M4 | | 27% |
| **Q6. When did you definitively decide on Emergency Medicine as a specialty?** | | |
| Prior to start of medical school | | 8% |
| M1 | | 2% |
| M2 | | 4% |
| M3 | | 50% |
| M4 | | 34% |
| I am still deciding on a specialty | | 2% |
| **Q7. Please check all previous experiences you had prior to final specialty selection.** | | |
| Shadowing Opportunity | 73 % | |
| Required Clerkship | 50 % | |
| Elective Rotation | 80 % | |
| Worked on Original Research in the Field | 22 % | |
| Published Research in the Field | 9 % | |
| Family Member in Field | 8% | |
| Mentor in Field | 53% | |
| **Q8. Rank the following in order of importance from 1-10 with 1 being most important for choosing your specialty. Only include the items that you consider important.** | | |
| Variety in clinical encounters | 2.33 | |
| Work life balance | 2.74 | |
| Perceived job satisfaction | 2.77 | |
| Patient population served | 3.68 | |
| Intellectual compensation | 3.76 | |
| Demands on family | 4.68 | |
| Opportunity to teach | 4.78 | |
| Financial compensation | 4.98 | |
| Opportunity to do research | 7.15 | |
| Professional prestige | 7.44 | |
